# Rapid Photodegradation of Methyl Orange (MO) Assisted with Cu(II) and Tartaric Acid

**DOI:** 10.1371/journal.pone.0134298

**Published:** 2015-08-04

**Authors:** Jing Guo, Xue Chen, Ying Shi, Yeqing Lan, Chao Qin

**Affiliations:** College of Sciences, Nanjing Agricultural University, Nanjing 210095, P.R. China; Old Dominion Univ., UNITED STATES

## Abstract

Cu(II) and organic carboxylic acids, existing extensively in soil and aquatic environments, can form complexes that may play an important role in the photodegradation of organic contaminants. In this paper, the catalytic role of Cu(II) in the removal of methyl orange (MO) in the presence of tartaric acid with light was investigated through batch experiments. The results demonstrate that the introduction of Cu(II) could markedly enhance the photodegradation of MO. In addition, high initial concentrations of Cu(II) and tartaric acid benefited the decomposition of MO. The most rapid removal of MO assisted by Cu(II) was achieved at pH 3. The formation of Cu(II)-tartaric acid complexes was assumed to be the key factor, generating hydroxyl radicals (•OH) and other oxidizing free radicals under irradiation through a ligand-to-metal charge-transfer pathway that was responsible for the efficient degradation of MO. Some intermediates in the reaction system were also detected to support this reaction mechanism.

## Introduction

Advanced oxidation processes (AOPs), which have superseded biological procedures proven to be ineffective for the treatment of some contaminated effluents under certain conditions, have been successfully demonstrated as efficient methods of degradation of organic pollutants [1–3]. In AOPs, hydroxyl radicals (·OH) and other oxidizing free radicals engendered from the reaction system can effectively oxidize organic pollutants into carbon dioxide, water, and inorganic acids.

The Fenton process is an advanced oxidation process that is widely applied to treat a variety of organic pollutants due to its high efficiency, simple operation, and low cost [4, 5]. Hydroxyl (·OH) radicals are produced while hydrogen peroxide (H_2_O_2_) is decomposed in the presence of ferrous ions. UV-vis irradiation improves the efficiency of the process. Recently, alternative techniques such as photocatalysis of the novel iron sources, and complexes of Fe(III) and carboxylate anions for the degradation of organic contaminants have also received considerable attention [6–11]. Zuo and Hoigne [6] noted that photolysis of Fe(III)-oxalato complexes could lead to the formation of hydrogen peroxide (H_2_O_2_), which could react with Fe(II) to further yield Fe(III) and a hydroxyl radical (·OH). Then, hydroxyl radicals could non-selectively mineralize azo dyes to carbon dioxide and water due to their high oxidation potential [12].

Cu(II) exists in natural environments and some waste soils and waters from the electroplating and smelting industries. Like Fe(III), Cu(II) can form a complex with organic carboxylic acid and has a lower oxidation state, Cu(I). Thus, it is hypothesized that in the presence of organic carboxylic acid, Cu(II), can also set up a photo-Fenton-like reaction with H_2_O_2_ produced in situ, generating Cu(I) and some active free radicals through a pathway of metal-ligand-electron transfer under irradiation, just as Fe(III)/oxalate does.

Garcia-Segura et al. [13] investigated the combination of Cu(II) and Fe(III) to improve the mineralization of phthalic acid by a solar photoelectro-Fenton (SPEF) process. They reported that Cu(II)-carboxylate complexes were easily removed with ·OH that resulted from the photo-Fenton-like reaction of Fe(III)-carboxylate species, accelerating the degradation of organic acids [13]. However, they did not mention Cu(II)-carboxylate complexes as a ·OH source. Our previous study on Cu(II)-carboxylate complexes mainly focused on the catalytic role of Cu(II) in the reduction of Cr(VI) by tartaric acid [14].

In this study, the photodegradation of methyl orange (MO) catalyzed by Cu(II) and tartaric acid was investigated at different initial pH values and concentrations of Cu(II), MO, and tartaric acid. MO was selected as the model organic pollutant in this paper because it is a typical azo dye. Azo dye, which contributes to ~70% of all dyes in industries such as textiles, foodstuffs and leather, is of particular concern because they are known to be mutagenic and carcinogenic [7, 9, 10, 15]. Cu(I) and ·OH in the reaction system were also examined to reveal the potential degradation pathway of MO. The role of Cu(II) as a catalyst for the degradation of azo dyes with light in the presence of organic acids has never before been reported.

## Materials and Methods

### 2.1 Materials

Methyl orange was obtained from Beijing Chemical Reagents Company (Beijing, China), and its stock solution (1000 mg/L) was prepared in deionized water. Cu(II) (50 mmol/L) was prepared by dissolving CuSO_4_•5H_2_O (s) (analytic grade, Shanghai Zhenxing Chemical Reagent Factory, Shanghai, China) in deionized water. The stock solution of tartaric acid (analytic grade, Shanghai Chemical Reagent Co., Ltd, Shanghai, China) with a concentration of 50 mmol/L was prepared in deionized water.

2,2’-Biquinoline, a characteristic reagent for Cu(I), was obtained from Sigma-Aldrich (Saint Louis, MO, USA). Tertiary butyl alcohol (TBA, Chengdu Kelong Chemical Reagent Factory, Chengdu, China) and L-histidine (L-H, Sinopharm Chemical Reagent Co., Ltd, Shanghai, China) were analytical grade and served as the radical scavengers to determine the production of ·OH and other oxidative free radicals in the reaction systems. The other chemicals used in this study were at least analytical grade and used without further purification. All of the stock solutions were stored in a refrigerator at 4°C in the dark prior to use.

All of the glassware used in the experiments were cleaned by soaking in 1 mol/L HCl for more than 12 h, and thoroughly rinsed first with tap water, then with deionized water.

### 2.2. Photochemical experiments

The photodegradation of MO was conducted in an XPA-7 photochemical reactor (Xujiang electromechanical plant, Nanjing, China) that was equipped with a magnetic stirrer, a device that controlled the temperature, and light sources including 100, 300 and 500 W medium pressure Hg lamps and a 500 W Xenon lamp. The light source was positioned inside a cylindrical Pyrex vessel surrounded by a circulating Pyrex water jacket to cool the lamp. A schematic diagram of the photochemical reactor was illustrated in our previous paper [16]. The light extensities at the position of the quartz tubes (reaction system) for the 100, 300 and 500 W medium pressure Hg lamps and the 500 W Xenon lamp were 12.7, 16.8, and 20.1 mw/cm^2^ (measured by a UV-A irradiation meter, Beijing Normal University, China) and 26 500 Lux (measured by a ST-80C illumination meter, Beijing Normal University, China), respectively.

For typical photocatalytic reactions, the required amounts of the stock solutions of MO, Cu(II) and tartaric acid were introduced into a 50 mL quartz tube, and the mixed solution was diluted with deionized water. NaOH (0.1 mol/L) and H_2_SO_4_ (0.1 mol/L) were adopted to adjust the initial pH to the desired values (2, 3, 4, 5, 6, 7 and 8), and the final volume of the solution was adjusted to 40 mL. Then, the reaction tubes with 40 mL of solution were placed into the photochemical reactor and stirred with a magnetic bar at 500 rpm during irradiation. The temperature was maintained by a thermostatic bath. Control experiments were also performed under the same conditions. All of the experiments in this section were performed in triplicate.

### 2.3 Analytical methods

At given irradiation time intervals, a 1 mL aliquot of sample was removed with a pipette and diluted to 10 mL with HAc-NaAc buffer (pH = 5). The MO concentration was immediately determined using a UV-vis spectrometer (Beijing Ruili Corp, UV-9100) at the characteristic λ_max_ of 464 nm.

Cu(I), an intermediate, was detected using 2,20-biquinoline that acted as the chromogenic agent, which was extracted with isoamyl alcohol [17]. The absorbance was measured at 545 nm (see Fig A in S1 Text for the adsorption curve).

A CyberScan pH2100 Bench Meter (Eutech Instruments) was used to measure the pH of the reaction solution after three-point calibration.

## Results and Discussion

### 3.1 Catalytic role of Cu(II) in the photodegradation of MO in the presence of tartaric acid

**Fig 1:**
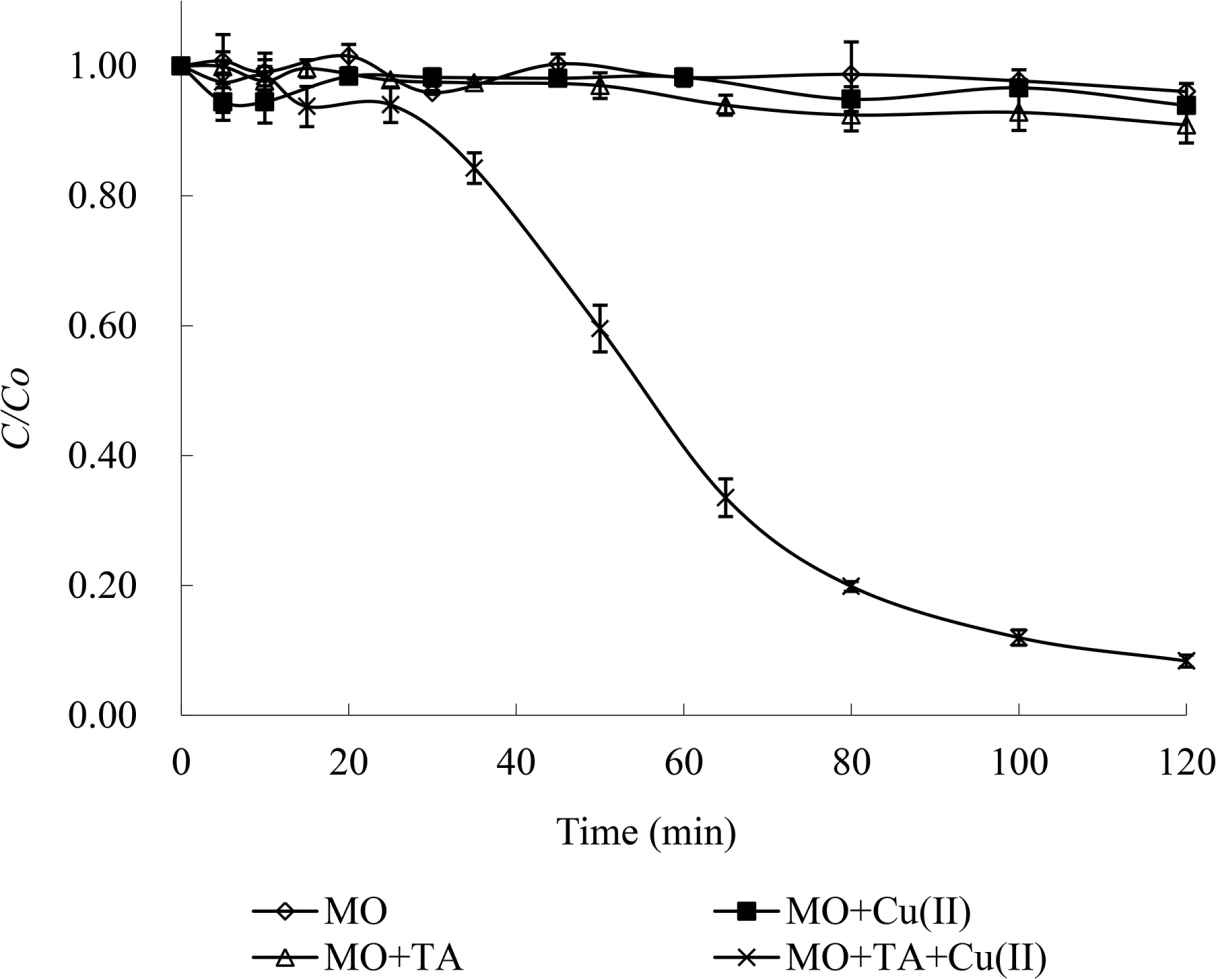
Photodegradation of MO in different reaction systems. Degradation conditions: 0.15 mmol/L MO, 1 mmol/L Cu(II) and 10 mmol/L tartaric acid (TA) under the full light of a 300 W medium pressure Hg lamp at pH 4 and 25°C.

The photodegradation of MO was conducted under different conditions. The results presented in Fig 1 show no noticeable change in the MO concentration in the single system of MO or the two-component system of MO and Cu(II) under UV irradiation with the full light of a 300 W medium pressure Hg lamp for 120 min, indicating that direct UV irradiation was insufficient to decompose MO even in the presence of Cu(II). A small increase in the MO degradation efficiency (~9%) in the two-component system of MO and tartaric acid was observed, which was attributed to the possible oxidants (e.g., H_2_O_2_ and some free radicals) that were produced through the photolysis of tartaric acid. A similar result was reported by Guo et al. [10], who investigated the photodegradation mechanism and kinetics of MO catalyzed by Fe(III) and citric acid. Possible reactions resulting in the removal of MO are described in Eqs (1–5).

$${\text{H}}_{2}\text{Tar}+{\text{O}}_{2}+hv\;\to \;{\text{H}}_{2}\text{Tar}\cdot^{+}+{\text{O}}_{2}\cdot^{-}$$ (1)

$${\text{H}}^{+}+{\text{O}}_{2}\cdot^{-}⇌{\text{HO}}_{2}\cdot$$ (2)

$${2\text{HO}}_{2}\cdot \;\to \;{\text{H}}_{2}{\text{O}}_{2}+{\text{O}}_{2}$$ (3)

$${\text{H}}_{2}{\text{O}}_{2}+hv\;\to \;2\cdot \text{OH}\;\left(\lambda <300\;\text{nm}\right)$$ (4)

MO+⋅OH→products (5)

However, 0.15 mmol/L MO was almost completely decolorized (the photodegradation efficiency reached 92%) within 120 min in the presence of 1 mmol/L Cu(II) and 10 mmol/L tartaric acid with UV irradiation, demonstrating that Cu(II) could significantly improve the photochemical degradation of MO in the presence of tartaric acid. The UV-vis spectra of MO versus time during the reaction are illustrated in Fig B in S1 Text, which clearly showed that the absorbance of the MO sample at the characteristic adsorption peak (464 nm) rapidly decreased with increasing reaction time.

It was reported that Fe(III) can strongly catalyze the degradation of MO by citric acid under weakly acidic conditions because of the formation of Fe(III)-citrate complexes, which is of high photocatalytic activity, to produce hydroxyl radicals through a photo-Fenton-like reaction system [8–10]. Similar to Fe(III), Cu(II) reacted with tartaric acid to form a complex and also had a lower oxidation state, Cu(I). Under the irradiation of light, the Cu(II)-tartaric acid complex produced Cu(I) and tartaric acid radicals through a pathway of metal-ligand-electron transfer (Eqs 6–8).

$$\text{Cu}\left(\text{II}\right)+\text{Tar}\to {\text{Cu}}^{\text{II}}\left(\text{Tar}\right)$$ (6)

$${\text{Cu}}^{\text{II}}\left(\text{Tar}\right)+hv\to {\text{Cu}}^{\text{I}}\left(\text{Tar}\right)\cdot$$ (7)

$${\text{Cu}}^{\text{I}}\left(\text{Tar}\right)\cdot \to \text{Cu}\left(\text{I}\right)+\text{Tar}\cdot$$ (8)

To prove this hypothesis, a full-length scan experiment was performed, demonstrating the formation of Cu(II)-tartaric acid complexes in the reaction system (data not shown). Furthermore, we introduced 2,2’-biquinoline into the reaction system, and a red complex in an isoamyl alcohol solution as an extracting agent was observed. The absorbance at λ_max_ = 545 nm was 0.093 (Fig A in S1 Text), which verified the generation of Cu(I) during the reaction [17] Moreover, tertiary butyl alcohol (a ·OH-specific radical scavenger) and L-histidine (a universal radical scavenger) [18] were introduced into the reaction systems. It was expected that these scavengers would serve to determine the production of ·OH and other oxidative free radicals in the reaction systems, and then the contributions of different radicals to the decomposition of MO could be discerned.

**Fig 2:**
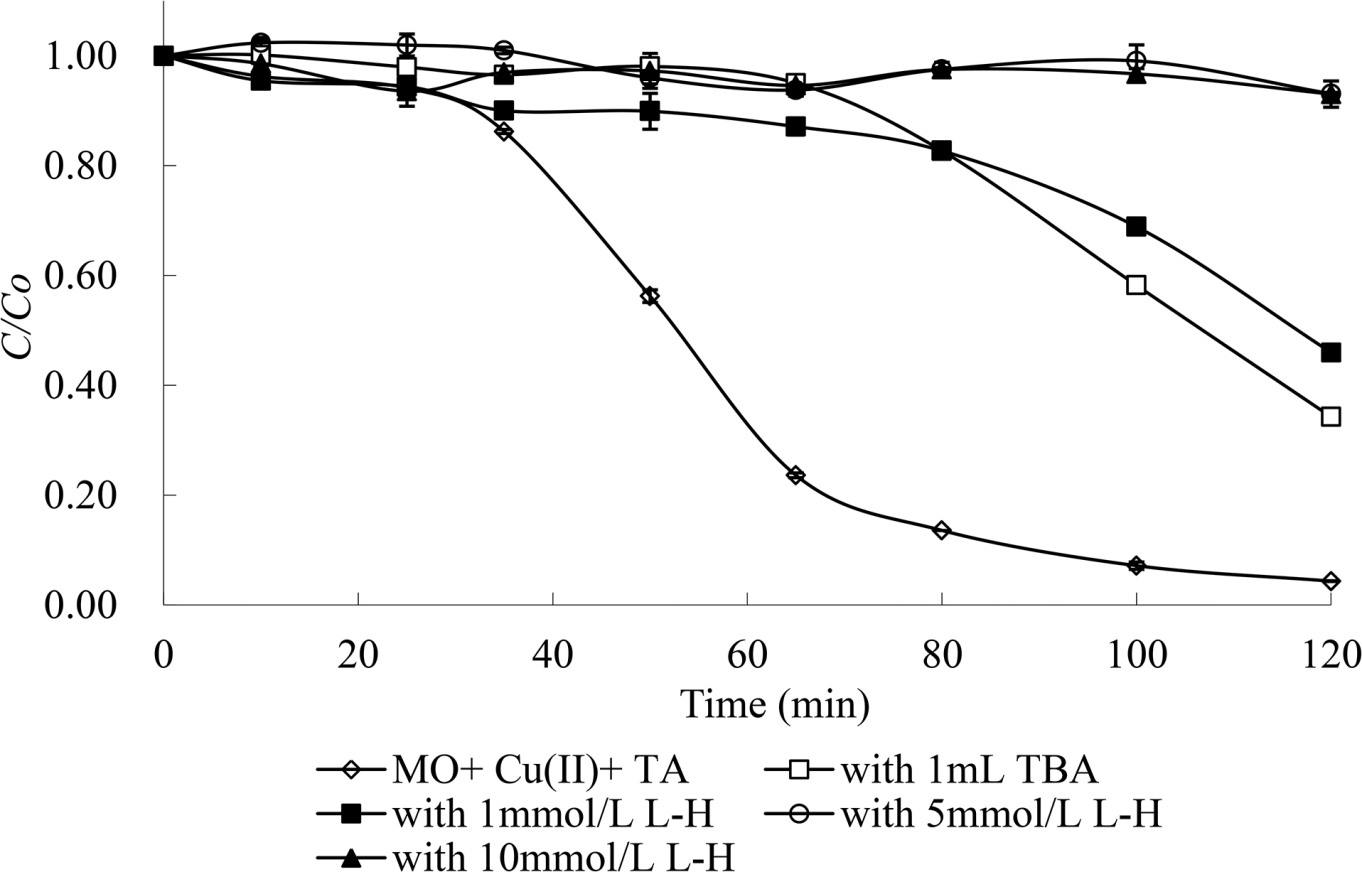
Effects of tertiary butyl alcohol (TBA, ~250 mmol/L) and l-histidine (l-H,) on MO degradation. Degradation conditions: 1 mmol/L Cu(II) and 10 mmol/L tartaric acid under the full light of a 300 W medium pressure Hg lamp at pH 4 and 25°C.

It was noted from Fig 2 that the Cu(II) catalytic degradation of MO in the presence of tartaric acid was clearly suppressed with the introduction of excess tertiary butyl alcohol (~250 mmol/L), especially in the initial 65 minutes (almost no MO degradation), confirming that ·OH resulted from the reaction system and contributed to MO decomposition to some degree. However, there was some MO (~64%) that was still degraded in the system containing excess tertiary butyl alcohol after 65 min, which indicated that there likely were some other types of active substances that were responsible for the destruction of MO.

L-histidine, which served as a scavenger of all of the oxidative free radicals in the reaction systems, was adopted in the Cu(II) catalytic degradation of MO in the presence of tartaric acid. The results (Fig 2) demonstrate that the photodegradation efficiency of MO decreased by 38% with the introduction of 1 mmol/L L-histidine within 120 min compared with that in the absence of scavengers, and the photodegradation was almost inhibited when the concentration of L-histidine was increased to 5 or 10 mmol/L. The results indicate that the degradation of MO involving radicals was almost completely quenched after the introduction of enough L-histidine. Based on the results described above, it was concluded that in addition to ·OH, there were some other types of free radicals that played an important role in MO decomposition. Moreover, these results corroborate the radical-based mechanism responsible for the destruction of MO with light in the presence of both Cu(II) and tartaric acid. The possible reactions apart from Eq (5) that were involved in the photodegradation of MO in the system of MO/Cu(II)/tartaric acid are summarized as follows:

$${\text{Cu}}^{\text{I}}\left(\text{Tar}\right)\cdot \overset{\;{\text{O}}_{2}\;}{\to }{\text{Cu}}^{\text{II}}\left(\text{Tar}\right)+{\text{O}}_{2}{}^{-}\cdot$$ (9)

$${\text{HO}}_{2}\cdot /{\text{O}}_{2}{}^{-}\cdot +\text{Cu}\left(\text{I}\right)\to \text{Cu}\left(\text{II}\right)+{\text{H}}_{2}{\text{O}}_{2}$$ (10)

$${\text{H}}_{2}{\text{O}}_{2}+\text{Cu}\left(\text{I}\right)\to \text{Cu}\left(\text{II}\right)+\cdot \text{OH}+{\text{OH}}^{-}$$ (11)

**Fig 3:**
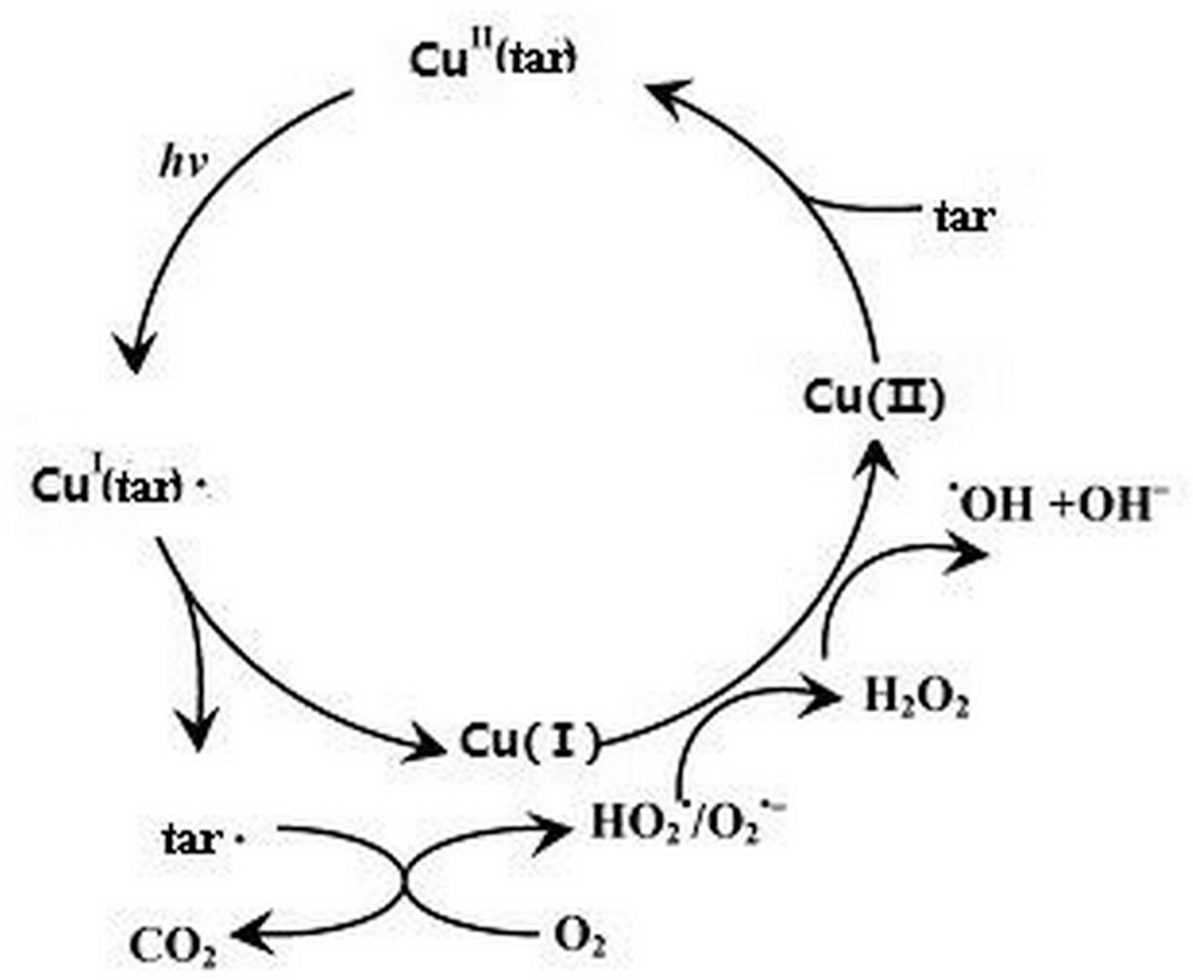
Scheme for Cu cycling and the main reactions in the Cu(II)-TA system.

During the generation process of these free radicals, Cu(I) and Cu^I^(tar)•, which were produced from the Cu(II)-tartaric acid complexes through a pathway of metal-ligand-electron transfer (Eqs 6–8), were oxidized by the oxidizing free radicals and dissolved oxygen in the reaction system, accompanied with the reproduction of Cu(II) (Eqs 10 and 11). Again, Cu(II)-tartaric acid complexes formed. Consequently, a cyclic process of converting Cu(II) to Cu(I) in the reaction system was established. The main reactions previously discussed were summarized in a possible mechanism scheme (Fig 3) for Cu(II) and Cu(I) cycling in the Cu(II)-tartaric acid system.

### 3.2 Effect of the initial concentrations of tartaric acid and Cu(II) on the photodegradation of MO

**Fig 4:**
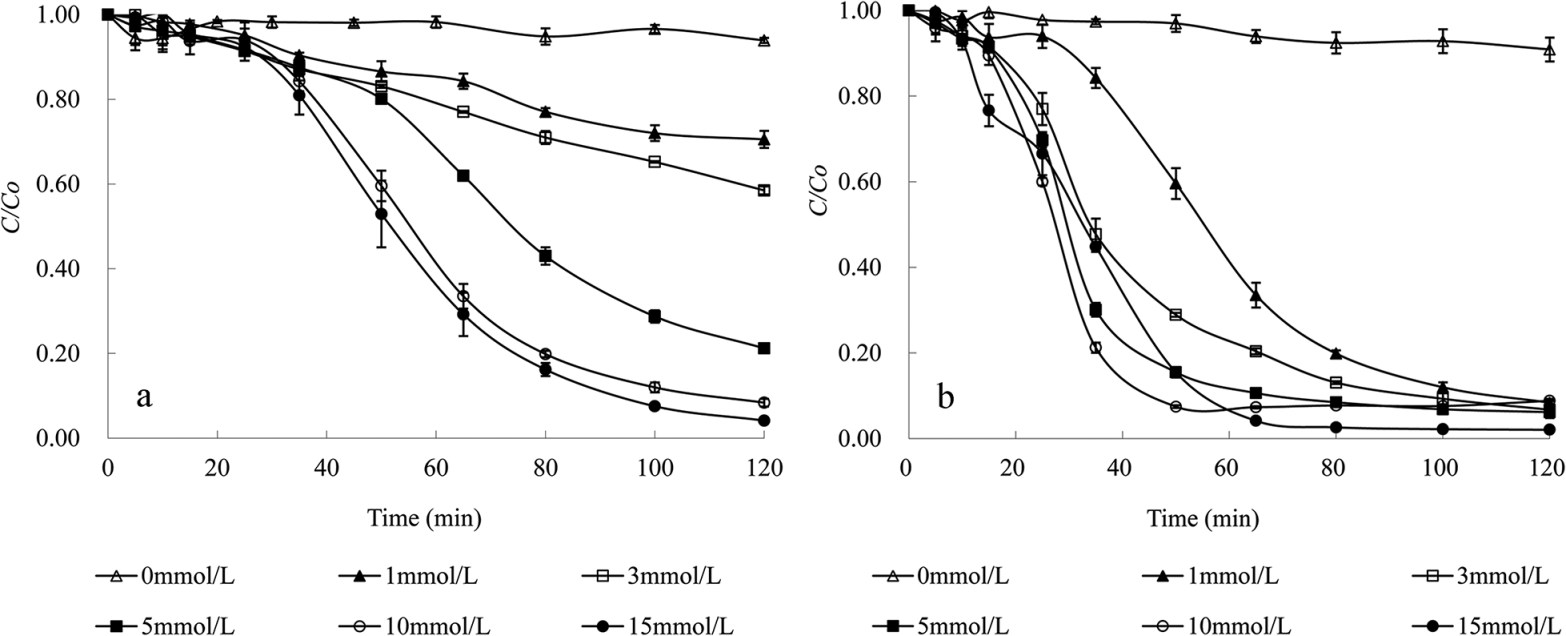
Effect of the initial concentrations of tartaric acid (a) and Cu(II) (b) on MO degradation. Degradation conditions: (a) with 1 mmol/L Cu(II), (b) with 10 mmol/L tartaric acid, under the full light of a 300 W medium pressure Hg lamp at pH 4 and 25°C.

The effect of the initial concentrations of tartaric acid and Cu(II) on the photodegradation of MO with irradiation by a 300 W medium pressure Hg lamp at pH 4 and 25°C was further investigated, and the results are depicted in Fig 4. As shown in Fig 4A, the increase of tartaric acid in the ternary system of MO/Cu(II)/tartaric acid with a given Cu(II) concentration greatly enhanced the photodegradation efficiency of MO. Similar results were reported by Balmer and Sulzberger [19] in an iron oxalate system, and they also noted that a higher oxalate concentration led to higher degradation of atrazine. The enhancement of the MO degradation by an increase in the tartaric acid concentration was due to the increased production of ·OH radicals and other active free radicals resulting from more the photochemically active Cu(II)-tartaric acid complexes that formed, as described in Eqs 6–11. Similarly, in the presence of 10 mmol/L tartaric acid, the degradation of MO significantly increased with the initial concentration of Cu(II) from 0 to 1 mmol/L (Fig 4B). This result suggested that the formation of Cu(II)-tartaric acid played a crucial role in generating ·OH radicals to accelerate the degradation of MO. However, as the initial concentration of Cu(II) increased from 1 to 15 mmol/L, there was no apparent enhancement in the MO degradation efficiency by the end of the reaction (120 min); however, there was an enhancement in the removal rate of MO in the beginning stage of the reaction. This result suggested that 1 mmol/L Cu(II) may be enough to form sufficient Cu(II)-tartaric acid complexes with 10 mmol/L tartaric acid to catalyze the photodegradation of 0.15 mmol/L MO.

### 3.3 Effect of pH on the photodegradation of MO

**Fig 5:**
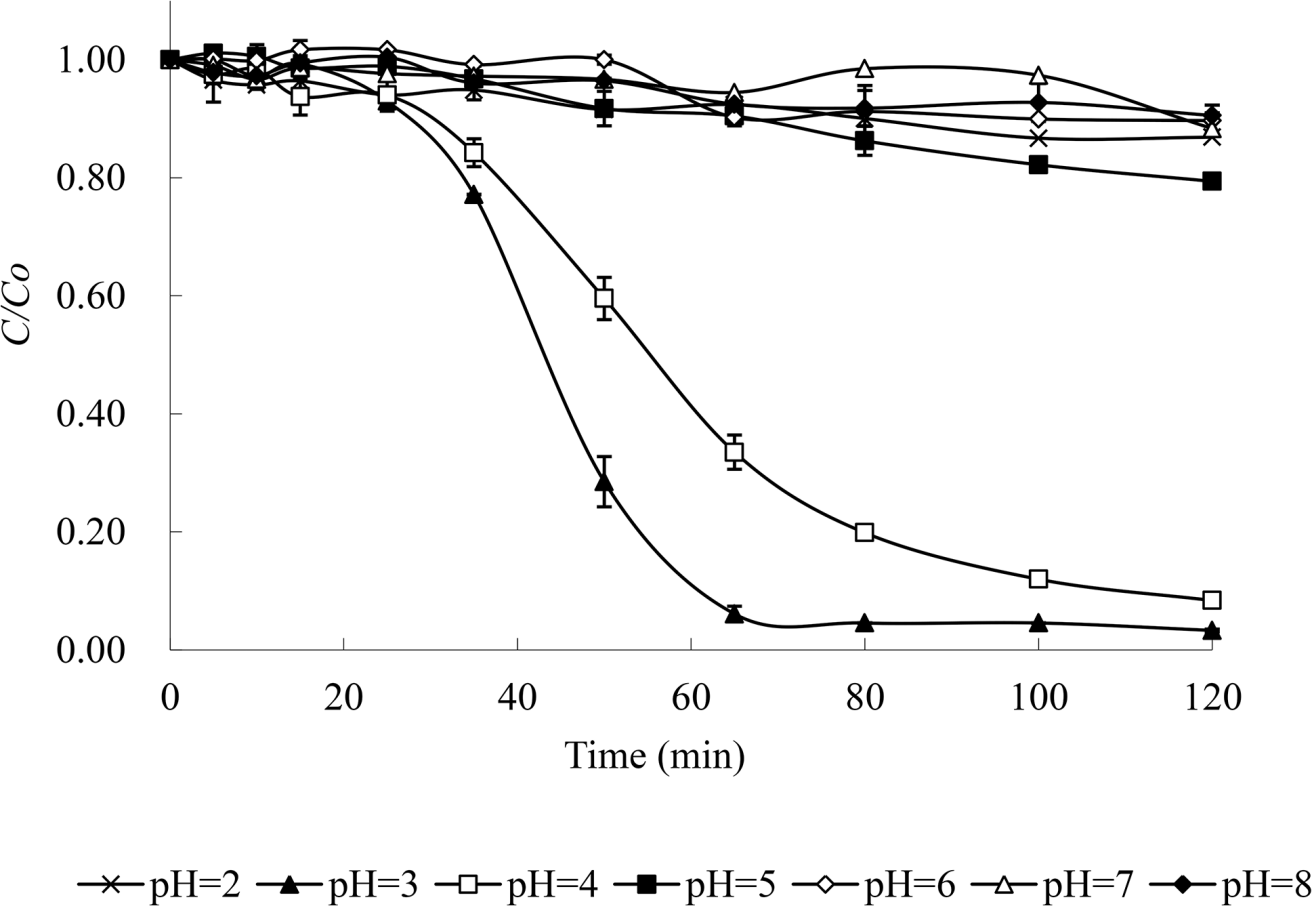
Effect of pH on the photodegradation of MO. Degradation conditions: 0.15 mmol/L MO, 1 mmol/L Cu(II) and 10 mmol/L tartaric acid under the full light of a 300 W medium pressure Hg lamp at 25°C.

Fig 5 shows the effect of pH on the photodegradation of MO in the presence of Cu(II) and tartaric acid. It was noted that pH played an important role in the degradation of MO, and the most efficient degradation of MO was realized at pH 3. In this case, MO was almost completely degraded within 70 min. Except for pH 3 and 4, the degradation rates of MO at pH 2 and 5–8 were similar and low, and less than 20% of the initial MO was removed within 120 min.

The dependence of the MO photodegradation on pH was considered to mainly result from the following factors. Firstly, high pH (pH 5–8) gave rise to an increase in Cu(II) hydrolysis, which had a negative effect on the formation of Cu(II)-tartaric acid complexes and did not benefit the photodegradation of MO. Secondly, it was supposed that low pH that benefited ·OH generation (Eqs 2 and 11) aided in MO photodegradation. However, this explanation was not true for the pH 2 condition. This was possibly due to the distribution of tartaric acid species and the quinoid structure of MO. Li et al. [18] reported that tartaric acid exists mainly as a molecular species at low pH, resulting in decreased Cu(II)-tartaric acid complexes. On the other hand, the molecular species of tartaric acid is prone to competing for ·OH with MO and degrades under UV light [20], which inhibits the photodegradation of MO. In addition, it is known that the structure of MO is quinoid at low pH (p*K*_a_ = 3.4), and the quinoid structure is more stable than the azo form [21]. Therefore, the degradation of MO was blocked at pH 2.

### 3.4 Effect of light intensity on the photodegradation of MO

**Fig 6:**
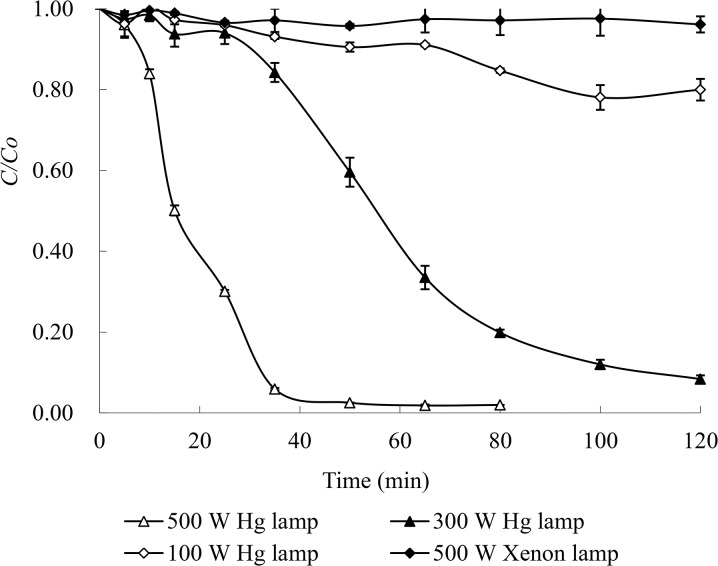
Effect of light intensity on the photodegradation of MO. Degradation conditions: 0.15 mmol/L MO, 1 mmol/L Cu(II) and 10 mmol/L tartaric acid at pH 4 and 25°C.

The photodegradation of MO under simulated solar light by a 500 W Xenon lamp and full ultraviolet light by 100 to 500 W medium pressure Hg lamps with an initial concentration of 0.15 mmol/L MO, 1 mmol/L Cu(II) and 10 mmol/L tartaric acid was investigated at pH 4 and 25°C. The results in Fig 6 show that the MO degradation under the irradiation of simulated solar light was negligible, indicating that solar light could not efficiently activate Cu(II)-tartaric acid complexes or tartaric acid to generate free radicals under this experimental condition. However, intensive ultraviolet irradiation could significantly enhance the MO degradation rate. Under the irradiation of 100, 300 and 500 W medium pressure Hg lamps, the MO degradation efficiency was 20% in 120 min, 92% in 120 min, and ~94% in 35 min, respectively. It was obvious that the photodegradation of MO strongly depended on light intensity in this system.

### 3.5 Improvement of the catalysis of Cu(II) and tartaric acid

**Fig 7:**
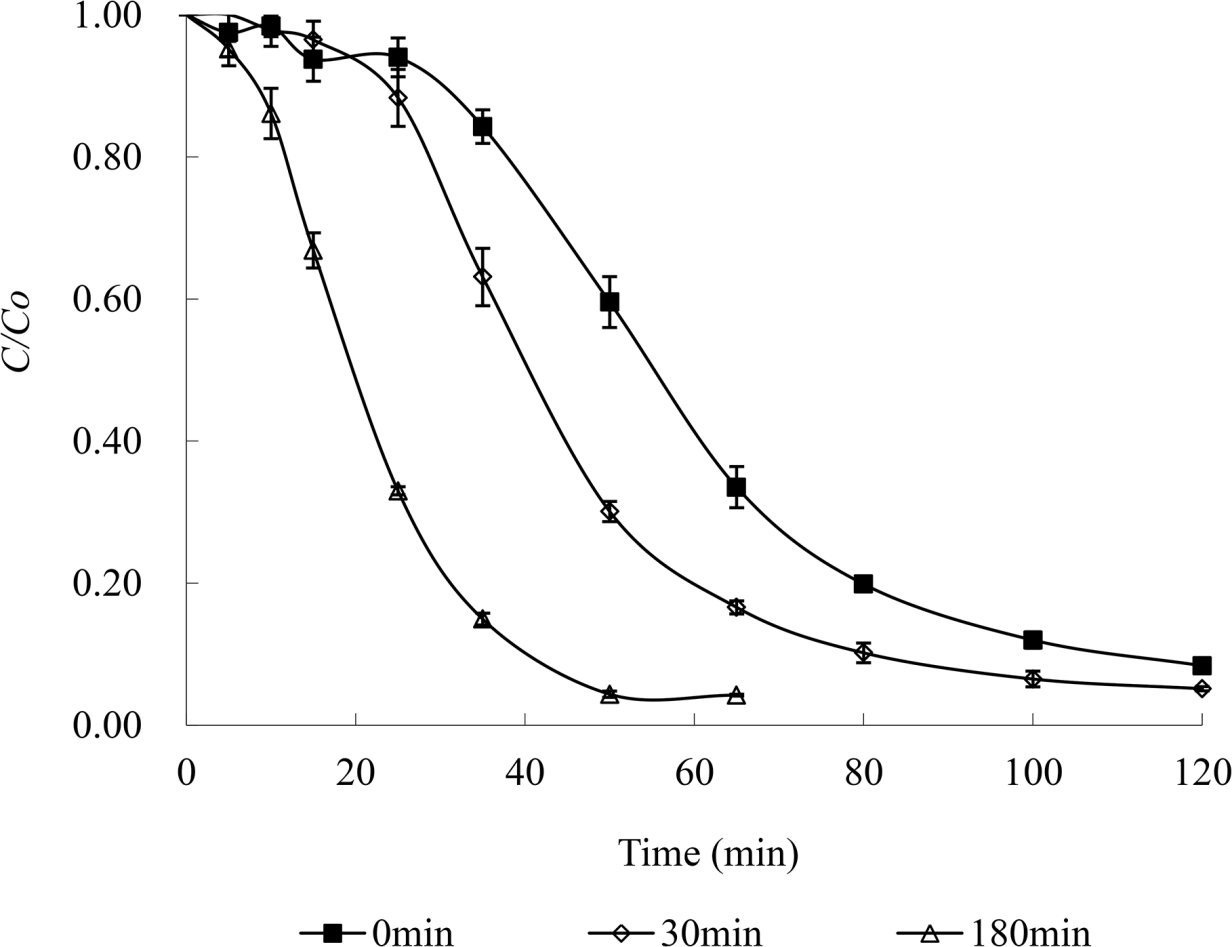
Photodegradation of MO by Cu(II)-tartaric acid with different pre-treatments. Degradation conditions: 0.15 mmol/L MO, 1 mmol/L Cu(II) and 10 mmol/L tartaric acid under the full light of a 300 W medium pressure Hg lamp at pH 4 and 25°C.

It was noted that the degradation rate was slow at the initial stage of the reaction and then accelerated after some reaction time in the ternary system of MO/Cu(II)/tartaric acid. It was assumed that the formation of Cu(II)-tartaric acid complexes required time. Thus, a series of experiments were carried out to prove this assumption. Cu(II) and tartaric acid were mixed for 30 min and 180 min with a magnetic stirrer in the photochemical reactor in the dark, and then MO was introduced into the reaction system to begin the experiment (the following steps were kept in routine operation). The results of MO degradation with different pre-treatments of Cu(II) and tartaric acid are shown in Fig 7. In comparison with the routine ternary system of MO/Cu(II)/tartaric acid without pre-treatment, when Cu(II) and tartaric acid were mixed in advance, the slow degradation stage in the beginning was shortened after 30 min of pre-treatment and almost disappeared after 180 min of pre-treatment. These results again illustrate that the formation of Cu(II)-tartaric acid complexes was the crucial step in the reaction system, and the photodegradation of MO could be accelerated by mixing Cu(II) and tartaric acid in advance.

## Conclusions

Cu(II) markedly catalyzed the photodegradation of methyl orange in the presence of tartaric acid under weakly acidic conditions. The degradation rate and efficiency was improved with an increase of the initial concentrations of Cu(II) and tartaric acid. The optimal degradation of methyl orange catalyzed by Cu(II) was achieved at pH 3. The formation of Cu(II)-tartaric acid complexes in the reaction system was the crucial step, from which the strong oxidizing agent (·OH) and other oxidizing free radicals were generated under irradiation by a medium pressure Hg lamp, accompanied by the cyclic process of Cu(II) to Cu(I) conversion. It could be inferred from this study that in natural environments or in some contaminated effluents with sunlight (including ~5% ultraviolet light), the transformation of Cu(II) to Cu(I) occurs when Cu(II) and organic carboxylic acid coexist, accompanied by the degradation of organic pollutants.

## Supporting Information

S1 Text Supporting information for “Rapid photodegradation of methyl orange (MO) assisted by Cu(II) and tartaric acid”. (DOCX)
