# Supplementary material for: Rapid Photodegradation of Methyl Orange (MO) Assisted with Cu(II) and Tartaric Acid
Source: PLoS One. 2015 Aug 4;10(8):e0134298. doi: 10.1371/journal.pone.0134298 (PMC4524705; doi:10.1371/journal.pone.0134298)
Supplement: S1 Text — (DOCX) [file pone.0134298.s001.docx]

# Supporting information for “Rapid photodegradation of methyl orange (MO) assisted by Cu(II) and tartaric acid”

Jing Guo, Xue Chen, Ying Shi, Yeqing Lan^*^, Chao Qin

College of Sciences, Nanjing Agricultural University, Nanjing 210095, P.R. China.


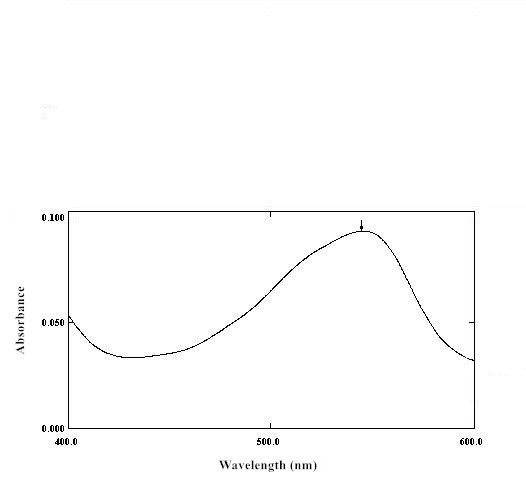


**Fig. A** The adsorption curve of the complex of Cu(I) and 2,2’-Biquinoline in isoamyl alcohol.


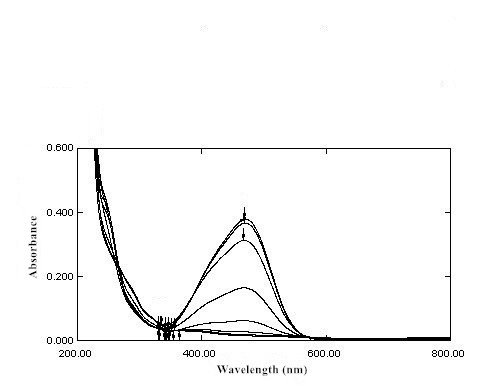


**Reaction Time (min)**

**0**

**20**

**30**

**45**

**60**

**80**

**100**

**120**

**Fig. B** UV-vis spectra of the photodegradation of 0.15 mmol/L MO catalyzed by 1 mmol/L Cu(II) and 10 mmol/L tartaric acid (TA) under the full light of a 300 W medium pressure Hg lamp at pH 4 and 25 ^o^C.
